# Carbon-Coated SiO_2_ Composites as Promising Anode Material for Li-Ion Batteries

**DOI:** 10.3390/molecules26154531

**Published:** 2021-07-27

**Authors:** Mihaela-Ramona Buga, Adnana Alina Spinu-Zaulet, Cosmin Giorgian Ungureanu, Raul-Augustin Mitran, Eugeniu Vasile, Mihaela Florea, Florentina Neatu

**Affiliations:** 1 National Research and Development Institute for Cryogenic and Isotopic Technologies Rm. Valcea, 4 Uzinei, 240050 Ramnicu Valcea, Romania; adnana.zaulet@icsi.ro (A.A.S.-Z.); cosmin.ungureanu@icsi.ro (C.G.U.); 2 Faculty of Power Engineering, Politehnica University of Bucharest, 060042 Bucharest, Romania; 3 “Ilie Murgulescu” Institute of Physical Chemistry, Romanian Academy, 202 Splaiul Indepedentei, 060021 Bucharest, Romania; raul.mitran@gmail.com; 4 Faculty of Applied Chemistry and Materials Science, Politehnica University of Bucharest, 060042 Bucharest, Romania; eugeniuvasile@yahoo.com; 5 National Institute of Materials Physics, 405A Atomistilor Street, 077125 Magurele, Romania; mihaela.florea@infim.ro (M.F.); florentina.neatu@infim.ro (F.N.)

**Keywords:** lithium-ion batteries, anode, silica, carbon coating, spray-coating

## Abstract

Porous silica-based materials are a promising alternative to graphite anodes for Li-ion batteries due to their high theoretical capacity, low discharge potential similar to pure silicon, superior cycling stability compared to silicon, abundance, and environmental friendliness. However, several challenges prevent the practical application of silica anodes, such as low coulombic efficiency and irreversible capacity losses during cycling. The main strategy to tackle the challenges of silica as an anode material has been developed to prepare carbon-coated SiO_2_ composites by carbonization in argon atmosphere. A facile and eco-friendly method of preparing carbon-coated SiO_2_ composites using sucrose is reported herein. The carbon-coated SiO_2_ composites were characterized using X-ray diffraction, X-ray photoelectron spectroscopy, thermogravimetry, transmission and scanning electron microscopy coupled with energy-dispersive X-ray spectroscopy, cyclic voltammetry, and charge–discharge cycling. A C/SiO_2_-0.085 M calendered electrode displays the best cycling stability, capacity of 714.3 mAh·g^−1^, and coulombic efficiency as well as the lowest charge transfer resistance over 200 cycles without electrode degradation. The electrochemical performance improvement could be attributed to the positive effect of the carbon thin layer that can effectively diminish interfacial impedance.

## 1. Introduction

Li-ion battery technology is at the core of the imminent massive vehicle electrification due to the forecast growth penetration rate of electrified vehicles [1]. In this cutting-edge Li-ion battery technology, silicon seems to be the most promising candidate for next-generation Li-ion battery technology due to its high theoretical capacity of 4200 mAh·g^−1^ (compared to 372 mAh·g^−1^ for graphite) [2,3]. Shifting to silicon as an anode material has the potential to deliver higher energy density for the batteries. The anode of a Li-ion battery should operate at low potentials and offer high specific energy capacity and density. However, several drastic challenges prevent the practical application of silicon anodes such as its huge (300%) volume change upon full lithiation, which causes the solid electrolyte interface (SEI) rupture or particle pulverization, leading to loss of electrical contact, fast reversible capacity loss, and low coulombic efficiency [2,4,5,6,7,8,9,10,11,12,13,14,15]. In recent years, research interest toward SiO_2_-based materials as a promising new alternative to graphite has been significantly increased due to the high theoretical capacity and low discharge potential similar to pure silicon. Research groups all over the world studied intensively how to tackle the drawback of low coulombic efficiency and irreversible capacity losses during cycling [16,17,18,19,20,21,22,23]. A main strategy to overcome the challenges of silica as an anode material has been to use carbon as a conductive matrix material. Carbon coating provides an effective solution to the above issue, improving the cycling stability by buffering the volume expansion of the silica particles [24,25,26,27,28,29,30]. Therefore, it is desirable to design a sustainable and eco-friendly preparation route for C/SiO_2_ composites. Different complex preparation routes and sacrificial templates of carbon-coated porous silica composites were also reported as an anode for Li-ion batteries [31,32,33].

Herein, we report a facile preparation route of the carbon-coated SiO_2_ composites. Sucrose was employed as carbon source to prepare the carbon-coated SiO_2_ composites by carbonization in argon atmosphere. The electrochemical performance was studied by cyclic voltammetry and charge/discharge tests. Unlike most reported carbon silica composites, the method proposed herein does not make use of complex procedures involving toxic precursors or strong acids such as sulfuric acid, and it has yielded novel composites with high electrochemical stability. The effect of starting carbon source concentration on the physicochemical and electrochemical properties of the resulting materials were also investigated.

## 2. Results and Discussion

### 2.1. Materials Preparation

**Scheme 1:**
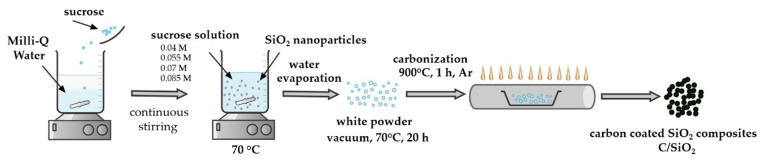
Schematic illustration of the preparation process of the carbon-coated SiO_2_ composites.

Carbon-coated SiO_2_ composites were prepared using modified literature recipes according to the references [28,29,30,31,32,33,34,35]. The carbon-coated SiO_2_ composites preparation route is illustrated in Scheme 1. In brief, silica nanoparticles were added to a sucrose solution of varying concentration followed by water evaporation and carbonization in argon at 900 °C.

### 2.2. Materials Characterization

**Figure 1:**
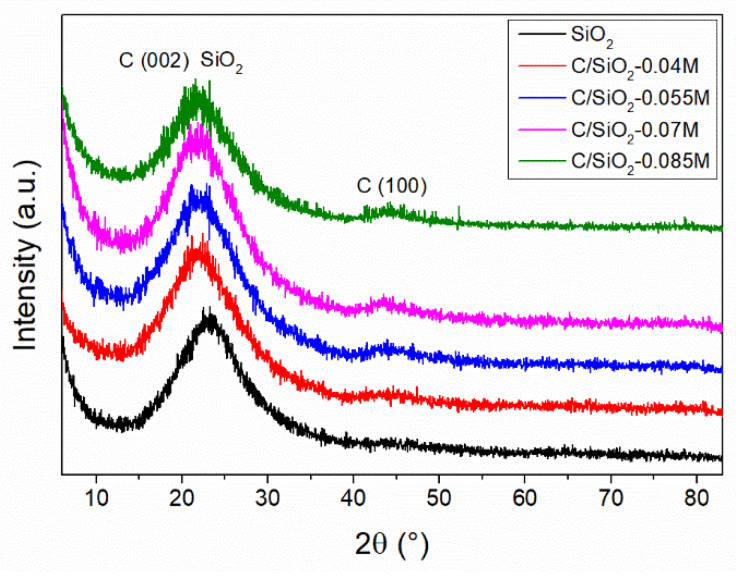
XRD patterns of silica and carbon–silica materials.

X-Ray Diffraction (XRD): The powder X-ray diffractograms were recorded for all samples (Figure 1). Silica shows only a single, broad peak, centered at 23.1° 2θ, corresponding to amorphous SiO_2_. In contrast, carbon–silica composites exhibit two broad diffraction peaks. The first broad peak is shifted to lower 2θ angles, which can be explained by the superposition of the amorphous silica and the (002) graphitic carbon reflection. The (100) graphitic carbon Bragg reflection can also be noticed at ≈43.7° 2θ for all carbon-containing samples. As expected, the relative intensity of the (100) reflection increases with increasing carbon content in the composite. Both carbon peaks are broad, indicating the nanometer size of the graphitic domains. However, the low intensity of the (100) peak and the superposition of the (002) reflection over the amorphous silica peak makes the exact determination of the crystallite size unreliable. No other peaks can be noticed, indicating that the composites contain only silica and carbon. 

**Figure 2:**
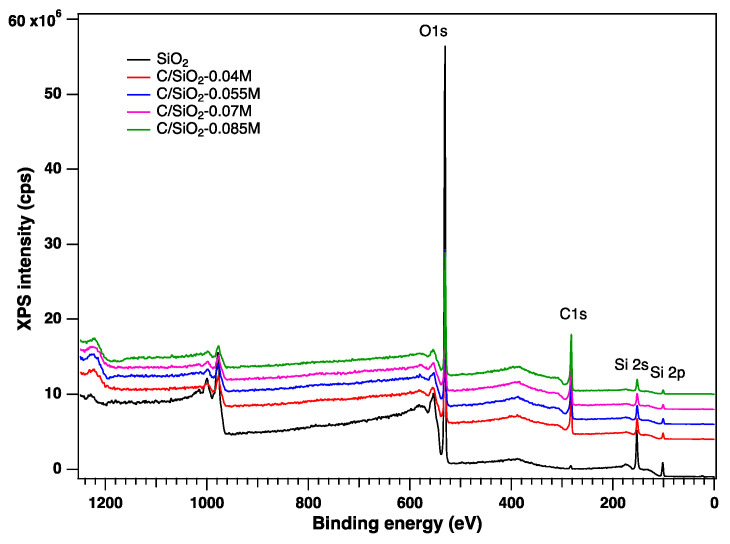
General XPS spectra of the studied samples.

**Figure 3:**
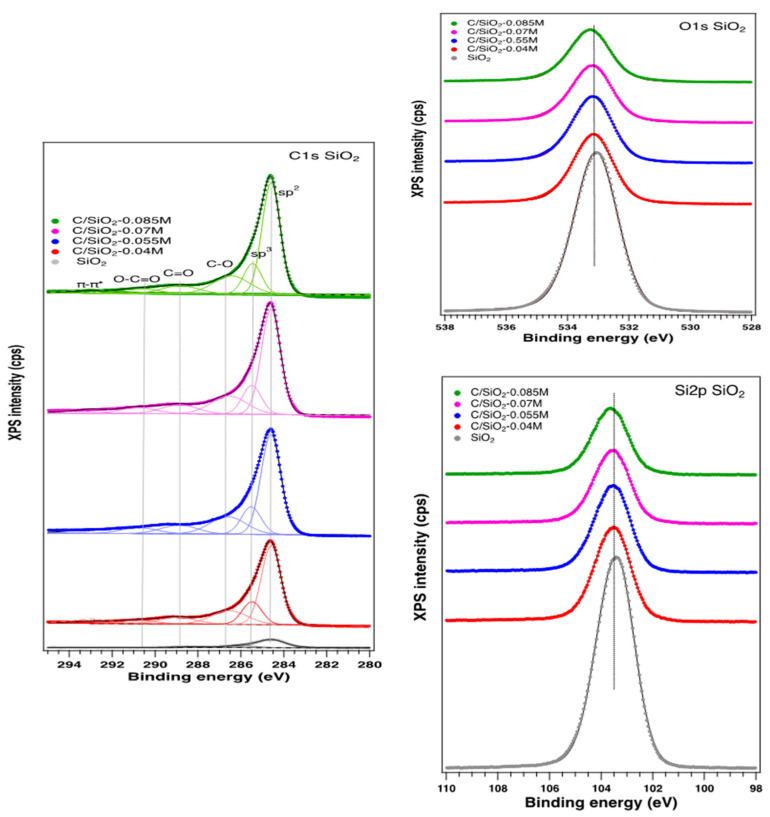
High resolution of C 1s, O 1s, and Si 2p for all the samples prepared.

X-Ray Photoelectron Spectroscopy (XPS): The surface chemical composition of the carbon–silica composite was determined by X-ray photoelectron spectroscopy (XPS). The general spectra, indicating the presence on the surface of Si, C, and O, are depicted in Figure 2. The C 1s, O 1s, and Si 2p high-resolution XPS spectra are presented in Figure 3. No significant changes in the O 1s and Si 2p spectra can be noticed, irrespective of the synthesis method. In contrast, the high-resolution C 1s peak can be fitted with several components, which depend on the preparation conditions. Thus, the components associated with C sp^2^ (284.6 eV), sp^3^ (285.5 eV), -C-OH (286.5 eV), O-C=O (288.8 eV), and -CO_2_ adventitious species (290.7 eV) were identified according with the literature data [36,37,38], as depicted in Table 1. The content in oxygenated carbon is low, in comparison with the C sp^2^ and sp^3^ which remain the main components (70%), denoting a good graphitization method. 

The carbon content on the surface is increasing with the initial sucrose solution concentrations but not in a constant manner. However, the concentration does not influence the content of different C species on the surface. It is interesting to note that the O content on the surface is decreasing with the sucrose solution concentrations, indicating a better graphitization and coverage of the surface with carbon, as confirmed by the other characterization methods [27]. The ratio between the O/Si is almost 2 in all cases, indicating a good consistency of the surface related to the bulk.

**Figure 4:**
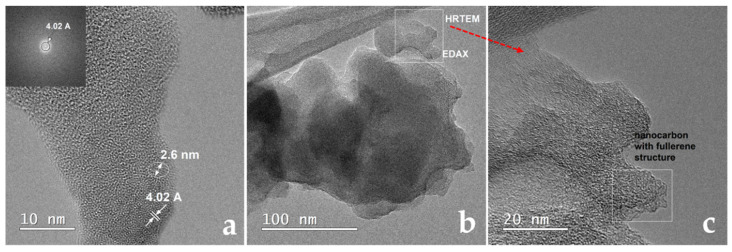
HRTEM image for the (**a**) SiO_2_; TEM image for the (**b**) C/SiO_2_—0.04 M at low magnification; (**c**) HRTEM image for the C/SiO_2_-0.04 M.

Transmission Electron Microscopy (TEM): TEM analysis was employed to assess the samples morphology and to confirm the XRD results (Figure 4). The high-resolution transmission electron microscopy (HRTEM, Figure 4a) image reveals an amorphous structure of the SiO_2_ sample, with atomic arrangements in rare crystalline clusters on small distances, below 2.6 nm. The distances between the parallel fringes indicate that the small ordered arrangements are around 4.02 Å. The Fourier transform (FFT) of the HRTEM image confirms the existence of a pseudo amorphous structure, the distances to the neighbors of 1st order being approximately 4 Å. The transmission electron microscopy (TEM) image in the light field (BF) (Figure 4b) shows an agglomeration of nanoparticles. Both the spaces between the SiO_2_ nanoparticles and the nanoparticles themselves are covered by a nanostructured carbon film (Figure 4c). The HRTEM image (Figure 4c) shows the nanostructure of the area highlighted by the square drawn in the image from Figure 4b. SiO_2_ nanoparticles are covered by a thin film consisting of carbon nanostructures, the ordered arrangements of atoms having very short range. Areas with “wormhole” morphology, which might contain fullerenic nanostructures can also be noticed (Figure 4c, marked area). The main element in the nanowire highlighted by the square drawn in the image in Figure 4b is carbon; also, the elements silicon and oxygen are present (EDAX spectrum—not presented).

**Figure 5:**
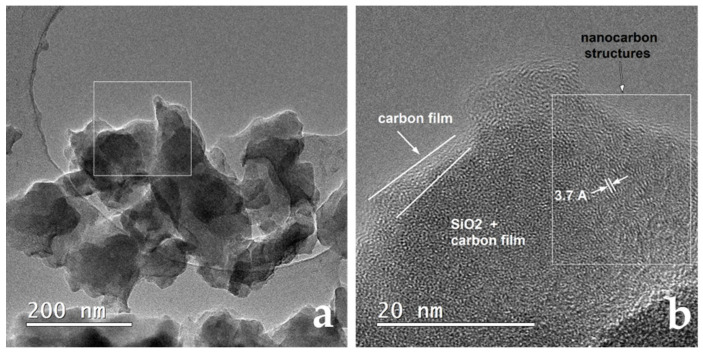
TEM image for the (**a**) C/SiO_2_-0.055 M at low magnification; HRTEM image for the (**b**) C/SiO_2_-0.055 M.

The transmission electron microscopy (TEM) image in the light field (BF) of the carbon-coated SiO_2_ composites with 0.005 M sucrose solution (Figure 5a) shows a large agglomeration of nanoparticles covered by a nanostructured carbon film. The high magnification image (Figure 5b) reveals a thin film consisting of carbon nanostructures, which exhibit ordered arrangement on short distances. The main elements in the area highlighted by the square in the image in Figure 5a are carbon, silicon, and oxygen (EDAX spectrum—not presented).

**Figure 6:**
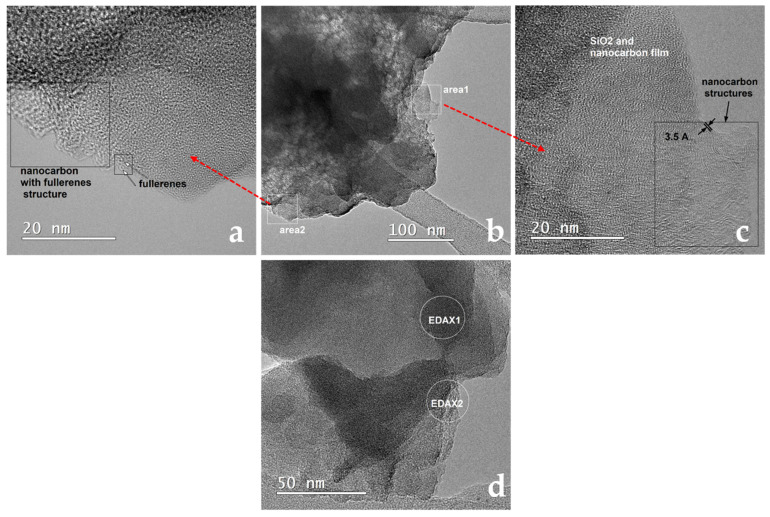
HRTEM image for the (**a**) C/SiO_2_-0.085 M—area2; TEM image for the (**b**) C/SiO_2_-0.085 M at low magnification; HRTEM image for the (**c**) C/SiO_2_-0.085 M area1; TEM image for the (**d**) C/SiO_2_-0.085 M.

The morphology of the SiO_2_-0.07 M sample is displayed in Figure 6. The light field TEM image (Figure 6b) exhibits a group of agglomerations of silica nanoparticles covered by an ultrafine size carbon nanostructured film. The HRTEM images shown in Figure 6a,c display carbon films of different morphologies: the film in “area1” is more compact, with denser nanostructures, while the film in “area2” has discontinuities, being composed of nanosphere-like formations. “Area1” HRTEM (Figure 6c) shows that the silica nanoparticles are covered by a thin film consisting of carbon nanostructures, the ordered arrangements of atoms being spaced at about 3.5 Å, which is a value commonly found in nanographitic structures. There are structures similar to short graphene strips but also possible turbostatic packaging. The HRTEM image in Figure 6a shows the nanostructure of the area marked with “area2”; in this case, in the carbon film is predominantly composed of fullerenic structures. The elemental composition was also verified by energy-dispersive X-ray nanoanalysis (EDAX). The EDAX spectrum (not presented) was acquired in the nano-zone marked with “EDAX1” in Figure 6d, proving the existence of silicon and oxygen as the main elements in the thicker areas (of darker contrast). The main element is carbon in the thin film surrounding the silica nanoparticles (the nano-zone marked “EDAX2” in Figure 6d.

**Figure 7:**
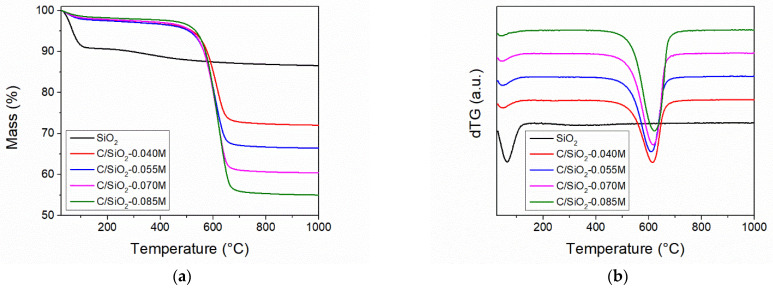
Thermogravimetric (**a**) and derivative thermogravimetry (**b**) analyses of the silica and carbon–silica samples.

Thermogravimetric Analyses (TGA): Thermogravimetric analyses were performed in order to assess the carbon and silica content of the samples as well as their thermal stability. All TG analyses were carried out in synthetic air atmosphere. All samples exhibit gradual mass loss up to ≈120 °C, which is ascribed to the loss of physisorbed water and residual solvent (Figure 7). The silica material shows a much higher content of physisorbed water in comparison with the carbon–silica composites, indicating that silica has a higher hydrophilic character than the composites. A gradual 4.7 wt % mass loss can be noticed for the SiO_2_ sample in the 200–900 °C temperature range, which is characteristic for the condensation of surface silanol groups. All carbon–silica composites present a sharp mass loss step between 450 and 700 °C (Figure 7b). This mass loss can be explained as the combustion of the carbon present in the samples, as carbon reacts with the oxygen, forming CO_2_. Based on the residual mass at 1000 °C, the sample composition could be computed (Table 2). The carbon content increases with increasing initial sucrose concentration, reaching 41.4 wt % carbon for the C/SiO_2_-0.085 M material.

**Figure 8:**
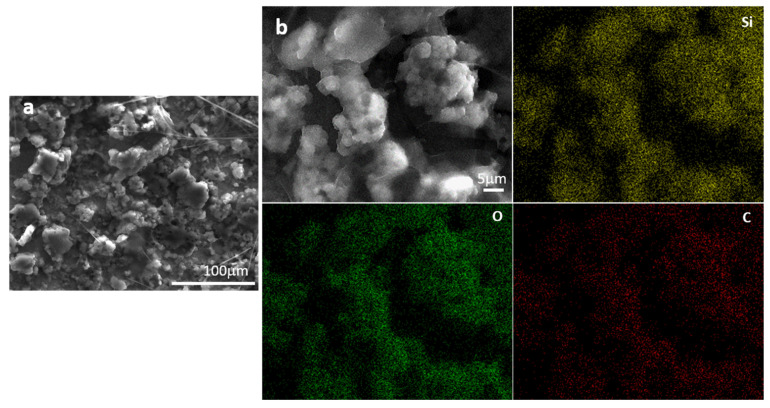
SEM image for the (**a**) C/SiO_2_-0.085 M calendered electrode after 200 cycles; (**b**) SEM-EDAX elemental mapping of Si, O and C of the C/SiO_2_-0.085 M calendered electrode after 200 cycles.

Scanning Electron Microscopy (SEM): The morphology and structure of the calendered C/SiO_2_-0.085 M electrode after 200 cycles is displayed in Figure 8. Good structural stability and no cracks were noticed after 200 cycles (Figure 8a). The carbon-coated layer could efficiently buffer the considerable volume expansion of SiO_2_ and prevent the electrode materials from pulverization during charge–discharge cycles. A homogenous distribution of Si, O, and C elements was observed (Figure 8b). The results confirm that silica nanoparticles were coated homogeneously with a carbon film that could explain the good electrochemical properties after 200 cycles.

### 2.3. Electrochemical Performance

**Figure 9:**
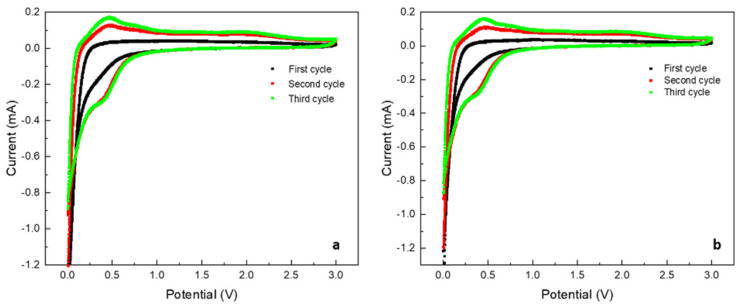
CV profiles of (**a**) uncalendered C/SiO_2_-0.085 M and (**b**) calendered C/SiO_2_-0.085 M, in the of 0.001–3 V voltage window, at a scan rate of 0.1 mV·s^−1^.

Cyclic voltammetry (CV) performance, for both uncalendered and calendered electrodes, was evaluated in the 0.001–3 V voltage window, at a scan rate of 0.1 mV·s^−1^ for the initial three cycles (Figure 9). Even though it is less visible in the first cycle, a cathodic peak appears between 0 and 0.5 V, which was attributed to the lithiation process of SiO_2_ for both samples [10]. The delithiation potential starts at 0.45 V and increases gradually until 1.15 V for the second and third cycles for the uncalendered electrode. The CV curves for the calendered C/SiO_2_-0.085 M electrode are shown in Figure 9b. Two anodic peaks can be observed at 0.45 V and 0.77 V at the second and third cycles, corresponding to the delithiation process [10]. All other materials exhibit similar voltage profiles with C/SiO_2_-0.085 M.

**Figure 10:**
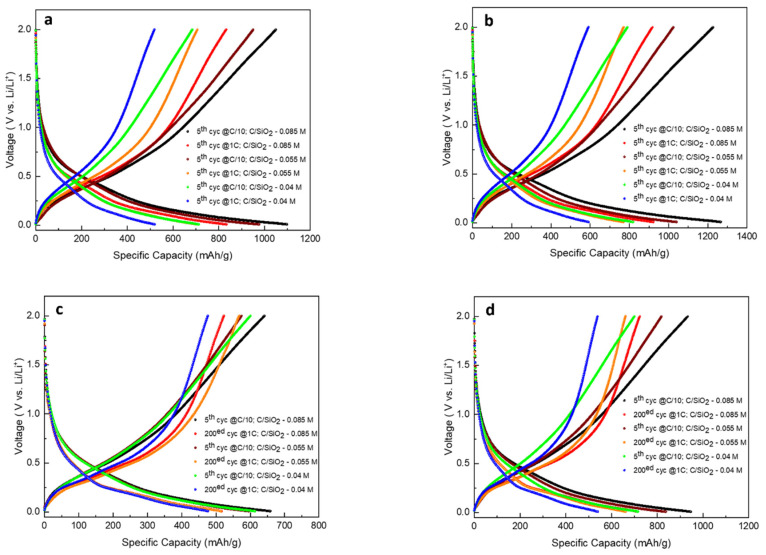
Charge–discharge voltage profiles of (**a**,**c**) C/SiO_2_-0.04 M, C/SiO_2_-0.055 M, and C/SiO_2_-0.085 M uncalendered electrodes (**b**,**d**) C/SiO_2_-0.04 M, C/SiO_2_-0.055 M, and C/SiO_2_-0.085 M calendered electrodes, at different cycles and C rates.

Figure 10 displays the charge–discharge voltage profiles of C/SiO_2_-0.04 M, C/SiO_2_-0.055 M, and C/SiO_2_-0.085 M uncalendered and calendered electrodes at different cycles and C rate. Calendered electrodes exhibit the best discharge capacities: the discharge capacities corresponding to the fifth cycles at C/10 for the C/SiO_2_-0.04 M, C/SiO_2_-0.055 M, and C/SiO_2_-0.085 M are 818.8 mAh·g^−1^, 1042.2 mAh·g^−1^, and 1231.3 mAh·g^−1^, respectively. It can also be seen that the calendered electrodes exhibit good discharge capacities at 1C: the discharge capacities corresponding to the fifth cycles at 1C for the C/SiO_2_-0.04 M, C/SiO_2_-0.055 M, and C/SiO_2_-0.085 M are 591.5 mAh·g^−1^, 765.1 mAh·g^−1^, and 922.1 mAh·g^−1^, respectively.

**Figure 11:**
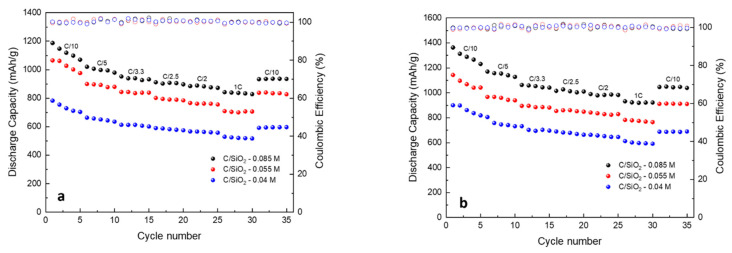
Rate capability of (**a**) C/SiO_2_-0.04 M, C/SiO_2_-0.055 M, and C/SiO_2_-0.085 M uncalendered electrodes and (**b**) C/SiO_2_-0.04 M, C/SiO_2_-0.055 M, and C/SiO_2_-0.085 M calendered electrodes, five cycles at C/10, C/5, C/3.3, C/2.5, C/2, 1C, and C/10.

The calendered electrodes show good rate capability, as presented in Figure 11. The C/SiO_2_-0.085 M composite’s calendered electrode shows the best rate performance (1231.3 mAh·g^−1^, coulombic efficiency (CE) = 99.7% at C/10 and 922.2 mAh·g^−1^, CE = 99.5% at 1C). An increasing capacity when increasing the carbon content can be noticed, which is in agreement with other results from literature. The capacity decreases at higher C rate; however, a higher specific capacity could be obtained when returned to C/10, indicating that the capacity decrease at high C rates is reversible.

**Figure 12:**
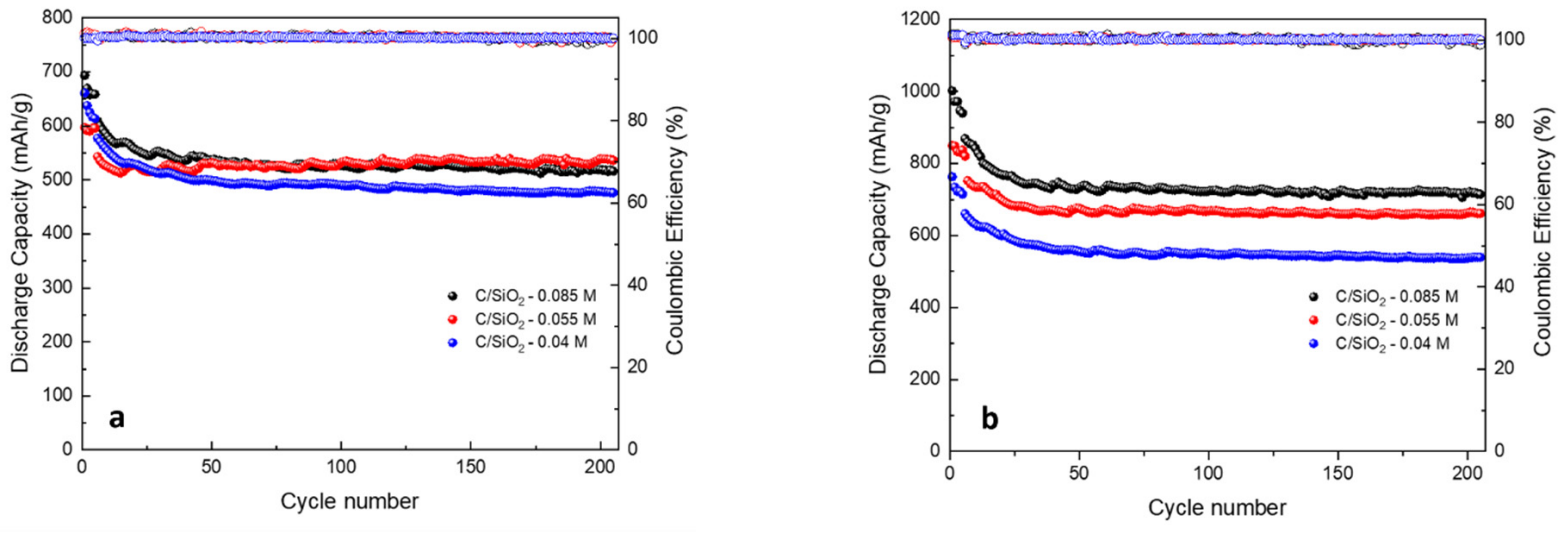
Cycling performance of (**a**) C/SiO_2_-0.04 M, C/SiO_2_-0.055 M, and C/SiO_2_-0.085 M, uncalendered electrodes and (**b**) C/SiO_2_-0.04 M, C/SiO_2_-0.055 M, and C/SiO_2_-0.085 M calendered electrodes, at 1C, 200 cycles.

Figure 12 displays the cycling performance of the C/SiO_2_ composites with different carbon contents for both uncalendered and calendered electrodes, at 1C, over 200 cycles. Best results were obtained for the calendered electrodes, while cycle life improved with the increase of the carbon content. The C/SiO_2_-0.085 M composite’s calendered electrodes (Figure 12b) show the best cycling performance after 200 cycles, the capacity remaining above 714.3 mAh·g^−1^ with CE = 98.9%). The results could be attributed to the coated carbon layer, which might act as a matrix that provides the best stable structure, buffering the volume changes during the cycling process. Increasing the carbon content could result in a thicker layer of coated carbon that can better accommodate the volume expansion of SiO_2_ during cycling.

**Figure 13:**
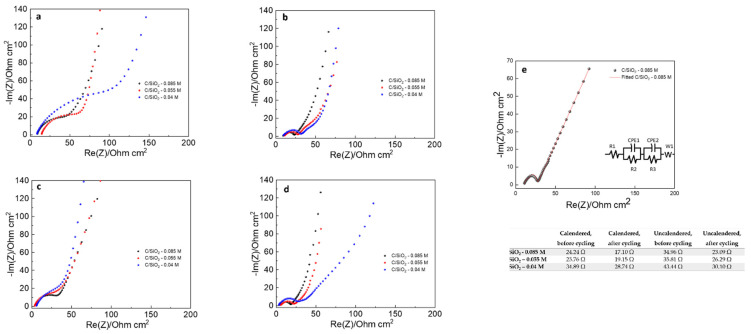
Electrochemical impedance spectroscopy of (**a**) C/SiO_2_-0.04 M, C/SiO_2_-0.055 M, and C/SiO_2_-0.085 M, uncalendered electrodes before cycling; (**b**) C/SiO_2_-0.04 M, C/SiO_2_-0.055 M, and C/SiO_2_-0.085 M, uncalendered electrodes after cycling; (**c**) C/SiO_2_-0.04 M, C/SiO_2_-0.055 M, and C/SiO_2_-0.085 M, calendered electrodes before cycling; (**d**) C/SiO_2_-0.04 M, C/SiO_2_-0.055 M, and C/SiO_2_-0.085 M, calendered electrodes after cycling; (**e**) The equivalent circuit of the C/SiO_2_-0.085 M uncalendered electrode after 200 cycles.

Electrochemical impedance spectroscopy (EIS) coupled with impedance fitting [39] was performed (Figure 13). The EIS spectra, represented in the form of Nyquist plots after 200 cycles (Figure 13a–d) for both uncalendered and calendered electrodes, displays smaller semicircles for both high and medium frequency, while the charge transfer resistance decreased. According to the literature, the Nyquist plots were fitted based on the equivalent circuit shown in Figure 13e, where R1 represents the bulk resistance of the electrolyte, R2 is associated with the contact resistance of the solid electrolyte interface (SEI) film, R3 is related to the resistance of the charge transfer, CPE1 is the charge capacitance 1, CPE2 is the charge capacitance 2, and W1 represent the Warburg diffusion element [9,10,28]. As presented in Figure 13, the charge transfer resistances for the uncalendered electrodes before cycling were 43.44 Ω for C/SiO_2_-0.04 M, 35.81 Ω for C/SiO_2_-0.055 M, and 34.96 Ω for C/SiO_2_-0.085 M, and after 200 cycles, they decreased to 30.10 Ω, 26.29 Ω, and 23.09, respectively. Regarding the calendered electrodes, lower values of the charge transfer resistances were obtained: before cycling 34.89.76 Ω for C/SiO_2_-0.04 M, 25.76 Ω for C/SiO_2_-0.055 M, and 24.24 Ω for C/SiO_2_-0.085 M, while after 200 cycles, 28.74 Ω, 19.15 Ω, and 17.10 Ω, respectively. Calendered electrodes exhibit lower values of the charge transfer resistance than the uncalendered ones. This may be due to the good contact of all electrode particles and good immersion of the electrolyte into the electrode [11]. The C/SiO_2_-0.085 M calendered electrode exhibits the lowest charge transfer resistance among all samples, suggesting that it could display better Li-ion transfer conductivity, which is in accordance with the results of the cycling performance.

## 3. Materials and Methods

### 3.1. Materials Preparation

All materials used in this study were of analytical grade and were used without further purification. The anode active materials—the carbon-coated SiO_2_ composites—were prepared via the aqueous pathway, which is a simply and eco-friendly preparation route. The preparation process of the carbon-coated SiO_2_ composites is illustrated in Scheme 1. Typically, aqueous sucrose (BioXtra, ≥99.5% (GC), Sigma-Aldrich) solutions (0.04 M, 0.055 M, 0.07 M, and 0.085 M) were prepared under continuous stirring. Then, the same amount of silica (Silicon dioxide nanopowder, spherical, porous, 5–15 nm in diameter, 99.5%, from Sigma Aldrich) was added to each sucrose solution. The resulting mixture was vigorous stirring at 70 °C until water was completely evaporated. The resulting white powder was dried in an oven, under vacuum, at 70 °C for 20 h, and then carbonized in a tube furnace in argon atmosphere at 900 °C for 1 h, using a heating ramp of 1 °C·min^−1^. Then, the prepared carbon-coated SiO_2_ composite materials with different carbon content were used to prepare the anodes.

### 3.2. Materials Characterization

X-Ray Diffraction (XRD) Analysis. SiO_2_ and carbon-coated SiO_2_ composite samples were characterized by X-ray diffraction (XRD). The XRD patterns were registered on a Panalytical X’PERT MPD Power Diffractometer (PANalytical, Almelo, The Netherlands) in the range 2θ = 10–80°, using Cu Kα radiation (λ = 1.5418 Å).

X-Ray Photoelectron Spectroscopy (XPS). An AXIS Ultra DLD (Kratos Analytical Ltd., Manchester, UK) set up was used to perform XPS measurements. The radiation used was a monochromated Al Kα (hν = 1486.7 eV) of an X-ray gun, operating at a power of 144 W (12 kV/12 mA), at normal emission, and on a routine base pressure of 1 × 10^−8^ Pa. To avoid charge effects, a charge neutralizer was used (electron acceleration at 1 eV and electron current of 0.1 mA) for all samples. The XPS spectra were fitted using a Voigt profile, and the spectra were calibrated at C1 s line (BE = 284.6 eV, C-C (CH)_n_ bonds) of the adsorbed hydrocarbon on the sample surface.

Transmission Electron Microscopy (TEM): The morphology and structure of the SiO_2_ and carbon-coated SiO_2_ composites samples was investigated through high-resolution transmission electron microscopy (HR-TEM) using a TECNAI–CM20 microscope equipped with an energy-dispersive X-ray analysis (EDAX) facility. 

Thermogravimetric Analyses (TGA). The thermal decomposition of samples was carried out using a Mettler Toledo TGA/SDTA851e thermogravimeter, at a heating rate of 10 °C·min^−1^, under 80 mL·min^−1^ synthetic air flow. All mass loss weight fractions were computed with respect to the dry sample mass, calculated at 150 °C.

Scanning Electron Microscopy (SEM). The morphology of the surface of the tested electrodes (after 200 cycles) was investigated through SEM analysis on a Quanta Inspect F instrument (FEI, Hillsboro, OR, USA), with a field emission electron gun, 1.2 nm resolution, and X-ray energy-dispersive (EDAX) spectrometer having an accelerating voltage of 30 kV.

### 3.3. Electrode Preparation

The electrode mixture was prepared by dispersing the active material (carbon-coated SiO_2_ composite) with carbon additive in an aqueous solution (Milli-Q Water) of Carboxymethyl Cellulose CMC (Daicel Miraizu Ltd., Tokyo, Japan) for 2 h using an ultrasonic processor. Then, Styrene Butadiene Rubber SBR (JSR Micro NV) was added and gently mixed for 20 min. The final electrode suspension containing 80 wt % active material, 3 wt % super P (MTI Corp.), and 17 wt % CMC/SBR was sprayed uniformly onto a copper current collector foil (15 µm thickness, from Custom Cells) using an airbrush (IWATA HP-BCS, 0.5 mm nozzle and needle). The electrode sheets were dried in vacuum at 70 °C overnight. Some samples were subsequently calendered using a 3 t Roll Press Equipment from Thank Metal Japan. Thirteen mm diameter circular electrodes—calendered and uncalendered—were punched using a compact precision disc cutter (MTI Corp., Richmond, CA, USA) and vacuum-dried overnight at 70 °C. The average mass of active material, after drying, was 0.18082 mg·cm^−2^. Electrode disks with similar loadings were transferred into an argon-filled glovebox for coin half-cells assembly (CR2032).

### 3.4. Electrochemical Performance

The CR2032 half-cells were assembled using 15.6 × 0.45 mm Li-metal chips (Xiamen TOB New Energy Technology CO., LTD) as the counter and reference electrode. Glass microfiber filter disks (Whatman GF/D, 19 mm in diameter) were used as separator. Then, 80 µL of 1 M LiPF_6_ in EC: EMC: DEC + 3 wt % FEC electrolyte supplied by Solvionic were used for each cell. The CR2032 cells were assembled using an electric coin cell crimper (MTI Corp.) and aged for 24 h. The charge–discharge measurements were carried out on a battery testing system (NEWARE) in a voltage range from 0.01 to 2 V. Cycling voltammetry measurements were recorded at room temperature using an Origaflex–OGF05A system, in the potential window of 0.001–3 V, at a scan rate of 0.1 mV·s^−1^. EIS measurements were performed before and after cycling, on a Solartron 1470 E Multi-Channel Potentiostat system over a frequency range from 100 KHz to 10 mHz.

## 4. Conclusions

Carbon-coated SiO_2_ composites were prepared through a facile and eco-friendly method, involving the carbonization of a sucrose–silica precursor at 900 °C. The effect of the starting sucrose concentration on the properties of the resulting materials was investigated in the 0.04–0.085 M range. All samples consist of carbon-coated silica nanoparticles. The carbon content increases with increasing precursor concentration. XPS analyses showed the presence of predominately sp^2^ hybridized carbon, with minor sp^3^ C and C-O species, while high-resolution TEM analyses have evidenced the presence of graphitized and fullerenic carbon nanofilms on the surface of the silica nanoparticles. 

Both calendered and uncalendered electrodes were prepared with the synthesized composites. Cyclic voltammetry measurements show that reversible lithiation and delithiation occurs for both types of electrodes. The discharge capacity of the calendered electrodes increases with increasing initial sucrose concentration at all C rates. The charge transfer resistance is inversely proportional to the initial precursor concentration while also being lower for the calendered electrodes. The transfer resistance is lower after 200 charging–discharging cycles in all cases.

The C/SiO_2_-0.085 M calendered electrode exhibits the best charging–discharging cycling stability after 200 cycles. No significant electrode degradation could be noticed. SEM-EDAX analyses on this electrode after 200 cycles show good structural stability and the absence of cracks, as well as the preservation of a homogenous distribution of carbon and silica. 

The C/SiO_2_-0.085 M material exhibits the best capacity of 714.3 mAh·g^−1^, coulombic efficiency near 100%, and the lowest charge transfer resistance, after 200 cycles, which can be attributed to the carbon-coated layer that effectively buffers the volume expansion of silica during the Li-ion insertion/extraction process. This material is a promising anode for high capacity and stability Li-ion batteries, which can be obtained through a facile and green method that does not use corrosive acids or solvents.

## Figures and Tables

**Table 1:** Binding energies of the present elements and their distribution.

Component	Binding Energy (eV)	SiO_2_ (%)	C/SiO_2_-0.04 M (%)	C/SiO_2_-0.055 M (%)	C/SiO_2_-0.07 M (%)	C/SiO_2_-0.085 M (%)
C 1s						
total		5	51	59	63	65
sp^2^	284.6	74	54	54	54	55
sp^3^	285.5	-	16	15	15	15
-C-OH	286.5	18	17	17	17	18
-O-C=O	288.8	8	8	9	9	8
CO_2_ adv	290.7	-	4	6	6	5
O 1s						
total		66	33	28	26	24
SiO	533.2	100	100	100	100	92
SiOH	534.4	-	-	-	-	8
Si 2p						
Si-O	103.7	29	16	13	11	11

**Table 2:** Sample composition based on TGA analyses.

Sample	SiO_2_ (wt %)	C (wt %)
SiO_2_	100.0	0.0
C/SiO_2_-0.040 M	77.2	22.8
C/SiO_2_-0.055 M	71.3	28.7
C/SiO_2_-0.070 M	64.7	35.3
C/SiO_2_-0.085 M	58.6	41.4

## Data Availability

Data is contained within the article.

## Sample Availability

Samples of the compounds SiO_2_, C/SiO_2_-0.04 M, C/SiO_2_-0.055 M, and 0.085 M are available from the authors.
